# Benchmarking Large Language Models for Sentiment Analysis in Pregnancy-Related Weight Discussions on Reddit: Human Performance-Aligned Comparative Evaluation Study

**DOI:** 10.2196/92821

**Published:** 2026-11-13

**Authors:** Rezaur Rashid, Brianna M White, Fekede Asefa Kumsa, Arash Shaban-Nejad

**Affiliations:** 1 Center for Biomedical Informatics, Department of Pediatrics, College of Medicine, University of Tennessee Health Science Center, 50 N Dunlap Street, Memphis, TN, 38103, United States, +1 (901) 287-5863

**Keywords:** gestational weight gain, body image, social media, sentiment analysis, large language models, Reddit, digital health, infodemiology

## Abstract

**Background:**

Pregnancy-related weight gain is both physiologically complex and socially charged, and online discussions often combine clinical information with implicit emotional distress. Although automated sentiment analysis could support research on pregnancy-related discourse, model performance in this context remains inadequately characterized relative to expert human judgment.

**Objective:**

This study aimed to benchmark contemporary sentiment classification models on pregnancy-related weight discussions, with emphasis on agreement with expert consensus, interpretive ambiguity, task adaptation, and deployment across posts and comments.

**Methods:**

We curated 15,619 Reddit posts and comments using a large language model (LLM)–guided keyword expansion strategy and established an adjudicated consensus reference set of 200 posts. We evaluated 10 models on a fixed held-out test set (N=150) using aligned text inputs, weighted performance metrics, and 1000-resample bootstrap CIs. RoBERTa (Robustly Optimized Bidirectional Encoder Representations from Transformers Approach) and BERTweet (Bidirectional Encoder Representations from Transformers for Tweets) were additionally evaluated in a 5-fold fine-tuning sensitivity analysis. Gemini-3-Flash (Google DeepMind [Google LLC]) and Generative Pretrained Transformer (GPT)–OSS (OpenAI) were then applied to 400 unlabeled posts and 15,019 comments with intermodel agreement assessment. To expand expert-labeled coverage, a separate 106-post annotation extension was used for secondary evaluation of the 2 models carried forward to post-level corpus inference, and cross-source recoverability of the API-derived retrieval was assessed using Arctic Shift (Arthur Heitmann).

**Results:**

The 2 primary experts (FAK and BMW) achieved 59.0% preadjudication agreement (Cohen κ=0.344). In the fixed held-out comparison, Gemini-3-Flash had the numerically highest weighted *F*_1_-score of 0.662 (κ=0.478), followed by GPT-OSS (*F*_1_-score=0.645; κ=0.436), with overlapping CIs across leading models. Under the harmonized direct-argmax decision rule with minimally processed text, BERTweet achieved an *F*_1_-score of 0.623 (κ=0.369), and RoBERTa achieved an *F*_1_-score of 0.605 (κ=0.339). In the separate fine-tuning sensitivity analysis, the mean cross-validated *F*_1_-score was 0.708 (SD 0.087) for RoBERTa and 0.616 (SD 0.079) for BERTweet. Among Gemini-3-Flash errors, 55.8% (29/52) occurred in posts on which the 2 primary experts disagreed. In the 106-post annotation extension, primary-expert agreement was 64.2% (68/106; κ=0.367); Gemini-3-Flash and GPT-OSS achieved weighted *F*_1_-score values of 0.633 and 0.680, respectively, and both identified 56 of 58 expert-negative posts in an exploratory human-review prioritization analysis. All 714 API-retrieved post IDs were independently recoverable through Arctic Shift. Intermodel agreement was 92.3% for posts (κ=0.797; n=400 valid pairs) and 46.3% for comments (κ=0.102; n=1500).

**Conclusions:**

No single model architecture was uniformly superior, and performance depended on the adaptation regime, annotation ambiguity, and discourse type. These findings define important boundary conditions for sentiment analysis of naturalistic maternal-health discourse: broad polarity classification can support corpus prioritization and hypothesis generation but is insufficient as a stand-alone measure of patient experience.

## Introduction

Pregnancy is a critical period characterized by physiological, psychological, and social changes, including the anticipation of gestational weight gain (GWG). GWG remains an underexplored aspect of maternal health, particularly with respect to individual emotional experience and peer support. As a complex biological phenomenon pivotal to pregnancy, GWG supplies energy reserves needed to support pregnancy-related physiological changes [1-4]. The 2009 US Institute of Medicine (IOM) guidelines recommend GWG based on women’s prepregnancy BMI: underweight women (BMI<18.5) should gain 12.5‐18 kg; normal weight (BMI 18.5‐24.9) 11.5‐16 kg; overweight (BMI 25‐29.9) 7‐11.5 kg; and obese (BMI≥30) 5‐9 kg [1].

Deviations from these guidelines have been associated with adverse health outcomes for both pregnant women and their infants [2,4,5]. Excessive GWG increases the risk of gestational hypertension [6-8], gestational diabetes [4,6], cesarean birth [6,8,9], postpartum hemorrhage [8], postpartum weight retention [10], and long-term obesity [11]. Conversely, inadequate GWG is associated with low birth weight (LBW) and preterm birth, both of which carry significant implications for neonatal morbidity and mortality [7,12-14].

The determinants of GWG encompass a broad range of demographic, physiological, psychological, and behavioral factors [1,15-17]. Health system characteristics, including provider knowledge, attitudes toward GWG counseling, perceived sensitivity of weight-related discussions, and limited time allocated for prenatal care, further complicate the delivery and effectiveness of weight management counseling during prenatal visits [18-26]. These intersecting factors underscore the need for a holistic and multidimensional understanding of GWG determinants that extend beyond traditional clinical parameters. Understanding the sentiment surrounding GWG is crucial for designing effective interventions that address both the physical and psychosocial challenges pregnant individuals face.

Psychosocial factors, including body image perception, play a critical role in shaping women’s experiences of GWG. Pregnancy-associated bodily changes often provoke complex emotional responses, influenced by cultural norms, weight-related stigma, and individual expectations around physical appearance [27]. Body image perception, satisfaction, and concerns, both before and during pregnancy, can significantly influence GWG trajectories, while postpartum weight retention further complicates long-term weight management and body image. The rise of digital communication has positioned social media platforms as important spaces for sharing health experiences, seeking advice, and expressing concerns [28]. These forums often serve as peer support networks that can profoundly influence attitudes, beliefs, and behaviors related to pregnancy weight management [28].

Sentiment analysis is a natural language processing (NLP) approach used to classify emotional tone or evaluative orientation in text [29-32]. Foundational work established supervised document-level polarity classification, unsupervised semantic-orientation methods, and systematic annotation of opinions and emotions [30-32]. In this study, we distinguish instruction-following generative large language models (LLMs) from encoder-only sentiment classifiers based on RoBERTa (Robustly Optimized Bidirectional Encoder Representations from Transformers Approach) and BERTweet (Bidirectional Encoder Representations from Transformers for Tweets) backbones [33-38]. These model groups support different deployment regimes: prompted inference without task-specific training and supervised adaptation when labeled data are available. Related methods have been used to examine public attitudes and health-related discourse on social media [39-44].

Pregnancy-related weight discourse is clinically and socially sensitive and can involve substantial interpretive complexity [45-47]. A post may combine a numerical account of weight change with reassurance-seeking, fear of complications, perceived judgment, or body-image dissatisfaction. Positive affect about pregnancy may coexist with distress about bodily changes or perceived loss of control, and advice-seeking may be expressed in factually neutral language even when concern is implicit [27,28]. These overlapping signals can reasonably support neutral, negative, or mixed interpretations when a single dominant label is required. Whether automated systems can reliably capture these nuances, and how their performance should be interpreted relative to human disagreement, remains an open empirical question with implications for digital health research. Although LLMs demonstrate strong general text-classification capabilities, their reliability in pregnancy-related weight-management discourse remains insufficiently characterized [44].

Recent work on subjective NLP annotation has emphasized that annotator disagreement may reflect legitimate differences in interpretation rather than only annotation error, and that aggregating annotations into a single majority label can obscure this variation [48-51]. These considerations are particularly relevant to sentiment and emotional-language annotation, where mixed affect and contextual ambiguity can support different judgments. Perspectivist approaches therefore encourage attention to annotator-level variation alongside aggregate labels rather than treating all disagreement as noise [48-51]. In this study, a consensus reference remains necessary for conventional accuracy-based benchmarking, but preadjudication disagreement is retained and analyzed as a complementary indicator of task ambiguity.

In this study, we present a formative benchmarking analysis of sentiment classification models applied to pregnancy-related weight discussions on Reddit (Reddit, Inc). We evaluate 10 models across 3 practical groups: commercial instruction-following LLMs, open-weight LLMs enabling local deployment, and encoder-only sentiment classifiers. The analysis characterizes the conditions under which these models agree, disagree, and diverge from expert judgment. Model performance is assessed against an expert-adjudicated consensus reference derived through stratified sampling of the relevance-screened corpus. The study contributes: (1) a fixed held-out comparison under aligned text inputs; (2) a human-disagreement analysis that treats adjudicated labels as a consensus reference rather than objective ground truth; (3) a separate task-adaptation sensitivity analysis for RoBERTa and BERTweet; and (4) an exploratory assessment of model concordance when inference is extended from initiating posts to peer comments. Collectively, the findings from these analyses provide formative guidance for the responsible evaluation and interpretation of automated sentiment classification in sensitive public-health discourse. Beyond model comparison, we therefore examine when broad sentiment usefully represents naturalistic patient-generated health discourse and when contextual complexity limits that representation, distinguishing limitations of sentiment classification from limitations of the underlying data.

## Methods

### Study Design

We used a multistage methodological framework comprising keyword-based data retrieval, expert-adjudicated consensus-reference development, model benchmarking, and exploratory application to the remaining unlabeled corpus (Figure 1).

**Figure 1.**
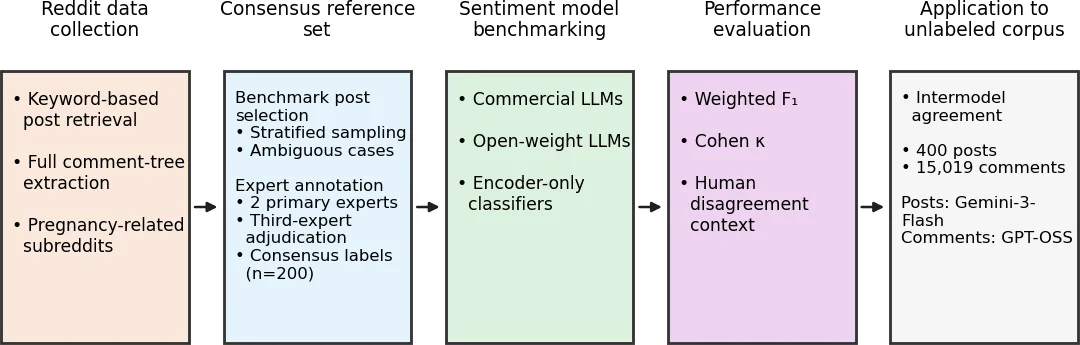
Overview of the benchmarking and deployment workflow. Reddit posts and associated comments were collected using keyword-based retrieval. A subset of posts was used to construct an expert-annotated benchmark reference set. Sentiment classification models spanning multiple architectural families were evaluated against this reference, and benchmarking results informed scalable inference on the remaining unlabeled corpus. GPT: generative pretrained transformer; LLM: large language model.

### Data Source and Keyword Generation

This study used data from Reddit, selected for its pseudonymous architecture, which facilitates self-disclosure of sensitive maternal health experiences. From a technical perspective, Reddit provides programmatic data access through its official API, whereas platforms such as X (formerly known as Twitter; Twitter Inc), and Facebook (Meta Platforms Inc) impose substantial cost barriers or have eliminated public API access entirely.

To capture the diverse linguistic registers used in online maternal health discussions, ranging from clinical terminology to informal expressions of distress, we used a 2-stage keyword curation process designed to bridge formal medical vocabulary and lay discourse. Initially, a domain expert (FAK) in GWG curated 29 evidence-based keywords (eg, GWG, BMI, postpartum weight retention, obesity and pregnancy, and pregnancy weight complications). Preliminary data extraction using this initial set of keywords (Textbox 1) yielded limited post retrieval (fewer than 100 posts), as these technical terms reflected the language in clinical practice guidelines and peer-reviewed literature, but did not align with the informal, experiential language predominantly used on Reddit.

Textbox 1. **Clinically curated seed keywords used for initial Reddit (Reddit, Inc) data collection. The list was developed by a maternal health domain expert and reflects terminology commonly used in clinical guidelines and the academic literature related to gestational weight gain (GWG) and pregnancy-associated body changes.**

maternal health, pregnancy weight, pregnancy weight complication, weight during pregnancy, weight gain during pregnancy, pregnancy weight gain stories, struggling with pregnancy weight, feeling fat during pregnancy, diet and pregnancy weight, body image during pregnancy, ashamed of pregnancy weight, pregnancy body, pregnancy weight changes, pregnancy body transformation, pregnancy weight fluctuations, weight management during pregnancy, pregnancy body confidence, feeling unattractive while pregnant, pregnancy weight insecurities, body shaming during pregnancy, pregnancy weight and mental health, pregnancy weight depression, pregnancy weight stigma, reality of pregnancy weight, cultural views on pregnancy weight, obesity and pregnancy, pregnancy body mass index, postpartum body confidence, bouncing back weight after birth

To address this limitation, we used GPT-4.5 to systematically expand the keyword set to include lay terminology, abbreviations, and informal phrases. The model was prompted as follows:

We are conducting research on maternal weight management discussions on Reddit. We have 29 clinical keywords [list provided]. Please expand this list to include colloquial terms, lay language, abbreviations, and common phrases that pregnant individuals might use on social media when discussing weight gain, weight management, and related concerns during pregnancy. Focus on terms that reflect authentic user language while maintaining relevance to pregnancy-related weight experiences.

This process generated 164 candidate terms (eg, baby weight, feeling fat, and pregnancy pounds). Following expert validation to remove irrelevant or overly broad terms, 144 keywords were retained (Supplementary Textbox S1 in Multimedia Appendix 1 contains the full list of expanded keywords). Illustrative examples of keyword expansion demonstrating the linguistic range captured across clinical, neutral, and emotionally expressive registers are shown in Textbox 2.

Textbox 2. **Examples of keyword expansion from clinical terminology to colloquial expressions. Clinical seed terms were expanded using an LLM-assisted (GPT-4.5) approach and subsequently filtered by a domain expert to capture informal language and experiential phrasing commonly used in Reddit discussions.**

**Clinical → colloquial**: “gestational weight gain” → “pregnancy weight gain” → “gaining baby weight” → “extra pounds during pregnancy” → “pregnancy pounds”

**Neutral descriptive**: “body image during pregnancy” → “pregnancy body changes” → “pregnancy body” → “pregnancy size”

**Experiential/emotional**: “weight management during pregnancy” → “weight struggles during pregnancy” → “struggling with pregnancy weight” → “feeling fat while pregnant” → “feeling overweight when pregnant” → “pregnancy weight issues” → “pregnancy weight problems”

**Support-seeking**: “pregnancy weight story” → “pregnancy weight journey” → “real pregnancy weight” → “honest pregnancy weight” → “pregnancy weight experiences” → “pregnancy weight diary”

**Distress/concern**: “pregnancy weight frustrations” → “pregnancy weight complaints” → “weight worries during pregnancy” → “pregnancy weight concerns” → “am I gaining too much weight during pregnancy”

### Data Collection and Preprocessing

Data collection was performed using the Python Reddit API Wrapper (PRAW, version 7.7.1; PRAW Development Team), which interfaces with Reddit’s official API. To focus retrieval on maternal health discourse, we restricted the search to 7 pregnancy- and women’s health-related subreddits selected a priori: r/pregnancy, r/babybumps, r/twoxchromosomes, r/womenshealth, r/bodyimage, r/obesity, and r/askreddit. Four subreddits contained posts meeting the keyword and relevance criteria and are represented in the final corpus. Because Reddit’s search and indexing behavior is not fully documented, the retrieved corpus should be interpreted as an API-derived sample rather than an exhaustive census. Ranking and indexing may favor more visible or recent content, while query and rate limits may truncate historical coverage.

Each of the 144 validated keyword phrases was submitted as a query to post titles and body text within the specified subreddits. The analysis code did not apply an additional case-sensitive filter, and topical screening was independent of capitalization. Searches had no preset date restriction; retrieved posts ranged from January 25, 2010, to April 7, 2025. Comments were not searched by keyword. Instead, nested comments and replies associated with each matched post were collected through PRAW comment-tree traversal. Metadata included unique identifiers, subreddit, text, Coordinated Universal Time (UTC) timestamps, upvote scores, and parent-child relationships among comments.

We retrieved an initial corpus of 714 unique posts. Three researchers (RR, BMW, and FAK) reviewed the retrieved posts using predefined relevance criteria. Posts were retained when they addressed pregnancy-related weight, body image, weight management, or weight-related emotional experience, and were excluded when they concerned nonpregnant weight loss, a partner’s weight, unrelated body image, deleted or URL-only content, or duplicates. Formal interrater agreement was not recorded for the relevance-screening stage; disagreements were resolved through discussion. The final analytical corpus contained 600 posts and 15,019 associated comments (Table 1).

**Table 1.** Descriptive statistics of the final analytical dataset^a^.

Subreddit	Number of posts	Number of comments	Number of unique users (total)	Average upvote score (posts)	Average upvote score (comments)
r/askreddit	8	88	58	1.63	1.41
r/babybumps	558	12,658	7468	32.15	5.16
r/twoxchromosomes	31	2249	1304	775.87	34.73
r/womenshealth	3	24	17	5.67	4.13

a The table summarizes the number of posts and comments, unique users, and engagement measures across included subreddits after preprocessing and exclusion criteria were applied.

### Independent Archive Sensitivity Analysis

As a cross-source sensitivity analysis, the 714 post-IDs identified during the original Reddit API retrieval were queried against Arctic Shift [52], an independent historical Reddit archive. Post-ID recovery was assessed as the primary measure, with subreddit, UTC creation timestamp, normalized title, and normalized post text compared as secondary consistency checks. This analysis was used to assess recoverability of the API-derived sampling frame and did not alter the original corpus or relevance-screening decisions.

### Data Preprocessing

For the primary comparison, all models received minimally processed post titles and body text. Casing, punctuation, URLs, emojis, and other stylistic cues were retained, with truncation only when required by a model-specific tokenizer limit. For RoBERTa and BERTweet, the primary held-out analysis used direct argmax over the native 3-class logits. Validation-calibrated thresholds were retained as a separate sensitivity analysis.

### Human Annotation and Consensus Reference Development

From the 600-post relevance-screened corpus, 210 posts were selected by stratified random sampling across the 4 contributing subreddits and temporal periods. The sampling frame and strata were defined from the relevance-screened corpus. The sample size was chosen to retain representation across those strata while remaining feasible for expert review. Adjudicated labels are treated as a consensus reference for evaluation, not as objective statements of a user’s emotional state.

Two primary experts (BMW and FAK) independently labeled all 210 posts for the overall emotional tone expressed by the original poster: positive, neutral, or negative. A third expert (RR) independently reviewed cases in which the primary experts disagreed. When the third label matched one of the primary labels, the majority label became the consensus reference. Ten posts (4.8%) received 3 different labels and therefore had no majority consensus. These posts were excluded from accuracy-based benchmarking, leaving 200 consensus-labeled posts, but were retained as maximal-ambiguity cases for qualitative discussion. Only posts were annotated because comments are context-dependent peer responses and were outside the original benchmark scope. Table 2 shows the consensus-label distribution.

**Table 2.** Sentiment-label distribution in the expert-adjudicated consensus reference set (N=200 posts)^a^.

Sentiment	Count, n (%)
Positive	49 (24.50)
Neutral	104 (52.00)
Negative	47 (23.50)

a Labels reflect consensus after adjudication and were used as the consensus reference for model benchmarking.

The annotation task instructed experts to classify the overall emotional tone conveyed by the original poster rather than the factual topic alone. The available study records document the 3 labels and this holistic instruction, but not a separately validated prospective emotion codebook or a formal calibration phase. The adjudication process therefore produced a usable consensus reference without eliminating the underlying interpretive ambiguity. The 10 three-way disagreements were not removed from the source corpus; they were excluded only from analyses requiring a single reference label and retained as maximal-ambiguity cases for qualitative interpretation.

### Interannotator Agreement

Prior to adjudication, the 2 primary experts (BMW and FAK) achieved 59.0% (124/210) raw agreement on all 210 posts (Cohen κ=0.344). We report the observed agreement directly, without assigning a categorical descriptor, because its purpose is to quantify the degree of interpretive disagreement present in the task.

### Annotation Extension

To expand expert-labeled coverage beyond the primary benchmark, 150 posts were sampled from posts not included in the original consensus reference using temporally stratified random sampling. Secondary expert relevance screening yielded 106 posts within the study scope. Two primary experts (BMW and FAK) independently classified the overall emotional tone of each retained post as positive, neutral, or negative using the same task definition as the primary annotation. Discordant labels were independently reviewed by a third expert (RR), and the majority label established the extension consensus reference. The extension was not used for prompt selection, threshold calibration, fine-tuning, or model selection.

### Model Selection and Categorization

We evaluated 10 models spanning 3 architectural families to evaluate trade-offs between reasoning capability, accessibility, and domain specificity (Supplementary Table S4 in Multimedia Appendix 1). This selection strategy mirrors recent benchmarking efforts in biomedical NLP by comparing general-purpose generators against specialized discriminative models.

1. Commercial instruction-following LLMs**:** We evaluated GPT-5-Mini (OpenAI), o3-Mini (OpenAI), and Gemini-3-Flash (Google). These models were selected as accessible, cost-efficient commercial systems representing realistic operating conditions for health-informatics research teams, rather than as frontier or state-of-the-art models.
2. Open-weight LLMs (local deployment and reproducibility): We evaluated LLaMA 3.1 (8B), Gemma 3 (12B), Mixtral (8 × 7B), OpenChat (7B), and GPT-OSS (20B). These models were deployed locally through Ollama v0.13.4 using 4-bit quantization to examine cost, privacy, and reproducibility trade-offs in a resource-constrained research environment.
3. Encoder-only sentiment classifiers**:** We evaluated RoBERTa (cardiffnlp/twitter-RoBERTa-base-sentiment-latest) and BERTweet (finiteautomata/BERTweet-base-sentiment-analysis) using Hugging Face Transformers v4.57.3. Unlike generative LLMs, these are encoder-only Transformer models with sentiment-classification heads adapted to social-media sentiment data. They were evaluated first as off-the-shelf sentiment classifiers under the aligned-input held-out design and then in a separate task-specific fine-tuning sensitivity analysis.

### Prompt Engineering

Effective prompt design is critical for eliciting accurate sentiment classifications from instruction-following language models, particularly in emotionally nuanced domains such as maternal health discourse. Following established best practices for LLM-based text annotation [53-55], we used a systematic, iterative prompt development process to optimize classification performance while minimizing subjective bias.

We iteratively tested prompt variations across multiple dimensions, including instruction phrasing (eg, “Classify.” vs “Determine.” versus “What is the sentiment.”), role framing (eg, “You are an expert in maternal health sentiment analysis” vs “You are a clinical researcher” vs no explicit role), etc. Each prompt variant was evaluated on a held-out 40-post validation subset drawn from the 200-post consensus reference to maximize alignment with expert annotations, with performance measured by weighted *F*_1_-score.

### Zero-Shot Prompt

For zero-shot inference, models were provided with a system persona and a direct classification task without examples. The final prompt was defined as follows:

You are an assistant trained to detect emotional sentiment in health-related online discussions. Analyze the following Reddit post, written by someone discussing weight gain during pregnancy. Assign a sentiment based on the emotional tone conveyed by the original poster: Positive, Negative, or Neutral. Respond with only one of the exact labels — no punctuation or explanation. Reddit post: {post_text}.

This prompt design constrains output to 1 of 3 labels without providing explicit sentiment definitions, allowing models to leverage their pretrained understanding of emotional tone while minimizing leading language that could bias classifications.

### Few-Shot Prompt Structure

#### Overview

To evaluate in-context learning, we tested 3 few-shot configurations: 1-shot, 3-shot, and 5-shot. Examples were sampled from a dedicated 10-post subset (separate from the 40-post validation set and 150-post test set) stratified by sentiment class to ensure balanced representation. For each target post classification, examples were randomly sampled according to stratified distributions: 1-shot prompts included 1 positive example; 3-shot prompts included 1 positive, 1 negative, and 1 neutral example (balanced representation); and 5-shot prompts included 2 positive, 2 negative, and 1 neutral example. The prompt structure followed an “Example-then-Classify” format:

You are an assistant trained to detect emotional sentiment in health-related online discussions. Analyze Reddit posts about pregnancy weight gain and classify the sentiment as: Positive, Negative, or Neutral. Here are some examples:

Example 1: Post: {example_1_text} Sentiment: {example_1_label}

Example 2: Post: {example_2_text} Sentiment: {example_2_label}

[. additional examples as needed .]

Now classify this post:

Post: {target_post_text}

Sentiment (one word only):

Each example was truncated to 1200 characters to standardize prompt length and avoid context window overflow, while preserving sufficient information for sentiment inference. Model outputs were parsed using a 2-stage extraction procedure to accommodate formatting variability. Case-insensitive regular expressions were first used to identify full sentiment labels (positive, negative, and neutral). When no full label was detected, common abbreviations (pos, neg, neu) were mapped to canonical forms. Outputs matching neither pattern, including refusals or ambiguous text, were labeled as Unknown and excluded from evaluation. Inference used deterministic decoding (temperature =0.0, max output token =10, where applicable) with a fixed random seed (seed=7) for few-shot example selection, ensuring reproducibility.

#### Performance Evaluation and Statistical Analysis

Performance on the fixed held-out test set (n=150) was evaluated with weighted *F*_1_-score as the primary metric and weighted precision, weighted recall, accuracy, and Cohen κ as secondary measures. For each model and metric, 95% CIs were estimated by nonparametric bootstrapping with 1000 resamples. Per-class *F*_1_-scores and confusion matrices were used to characterize error patterns. No pairwise superiority tests were performed; point estimates are therefore interpreted descriptively, with particular attention to the width and overlap of the bootstrap intervals.

For RoBERTa and BERTweet, the primary held-out comparison used direct argmax over the native 3-class logits to apply a uniform off-the-shelf decision rule. As a sensitivity analysis, class-specific thresholds were selected on the validation set through grid search from 0.30 to 0.90 in increments of 0.05 and then applied to the held-out test set. These calibrated results are reported separately from the primary benchmark. As a task-adaptation sensitivity analysis, RoBERTa and BERTweet were fine-tuned using stratified 5-fold cross-validation across the 200 consensus-labeled posts. Each fold used 160 posts for training and 40 for evaluation. Training used 4 epochs, a batch size of 8, a learning rate of 2×10⁻⁵, weight decay of 0.01, and random seed 7. Maximum sequence length was 256 tokens for RoBERTa and 128 for BERTweet. Results are reported as fold means and standard deviations and are not directly ranked against the fixed 150-post held-out estimates.

To contextualize automated model performance, we calculated the same metrics for human annotators relative to the adjudicated consensus labels. Expert-to-expert agreement quantifies task disagreement, while expert-to-consensus comparisons provide a separate human reference. Because these comparisons use different reference labels and sample sizes, they are not interpreted as a single numerical performance ceiling.

The annotation extension was used for secondary evaluation of Gemini-3-Flash and GPT-OSS, the 2 models carried forward to post-level corpus inference. Predictions for these posts had been generated before the extension consensus labels were established, and no additional prompt selection, threshold adjustment, or model adaptation was performed using the extension labels. Performance was summarized using weighted precision, recall, *F*_1_-score, accuracy, and Cohen κ with 1000-resample bootstrap CIs. For an exploratory human-review prioritization analysis, extension consensus labels were additionally collapsed to negative versus nonnegative (positive or neutral), and sensitivity, specificity, positive predictive value, negative predictive value, binary *F*_1_-score, and the proportion of posts flagged for review were calculated.

#### Application to Unlabeled Corpus

To demonstrate application to a larger unlabeled corpus, we used Gemini-3-Flash for the remaining 400 posts because it had the numerically highest weighted *F*_1_-score point estimate in the fixed held-out comparison. GPT-OSS was used for 15,019 comments because it had a similar held-out point estimate and supported cost-free local deployment for the larger corpus. These corpus-scale predictions are exploratory and are not treated as expert-validated sentiment prevalence estimates.

To examine consistency between the commercial and open-weight deployment choices, Cohen κ was calculated between Gemini-3-Flash and GPT-OSS for 400 unlabeled posts and a stratified random sample of 1500 comments (approximately 10% of the comment corpus). Intermodel agreement is interpreted as consistency, not accuracy, because neither unlabeled set has expert reference labels.

To examine whether corpus-scale sentiment classifications could identify substantive areas for follow-up analysis, we additionally compared valence-neutral pregnancy-weight discussion contexts among posts on which Gemini-3-Flash and GPT-OSS agreed. Ten nonexclusive contexts were defined using transparent lexical indicators (Supplementary Table S6 in Multimedia Appendix 1), with explicit sentiment-bearing terms excluded to reduce lexical circularity. Negative versus nonnegative context prevalence was compared using Fisher exact tests with Benjamini-Hochberg false-discovery-rate correction; model-specific analyses across all 400 posts were used as directional sensitivity checks. This analysis was exploratory and hypothesis-generating.

Comment-level findings require additional caution because model selection, prompt development, and expert validation were conducted on posts rather than comments. Differences between post and comment predictions also combine discourse-type effects with model effects because different models were used for the 2 corpora.

#### Ethical Considerations

This study was conducted within the scope of the University of Tennessee Health Science Center (UTHSC) protocol 23‐09294-NHSR, Perceptions and Misinformation Shared via Social Media Platforms: Sentiment Analysi*s*. The UTHSC Institutional Review Board determined that the research activities described under this protocol qualify as Not Human Subjects Research (NHSR). The protocol encompasses sentiment analysis of publicly available social media data, including Reddit, without access to direct personal identifiers or interaction with individuals. Usernames and other potentially identifying fields were excluded from the analytical data, and results are reported in aggregate to reduce reidentification risk.

## Results

### Overview

Results are presented in 3 stages: human agreement and task ambiguity, fixed held-out model performance with sensitivity and extension analyses, and exploratory application to the unlabeled corpus.

As a retrieval sensitivity check, all 714 post IDs identified through the original Reddit API retrieval were independently recoverable through Arctic Shift (714/714, 100%). Subreddit and UTC creation timestamp matched for all posts, normalized titles matched for 713/714 posts (99.9%), and normalized post text matched for 678/714 posts (95.0%).

### Human Expert Agreement and Task Difficulty

Before adjudication, the 2 primary experts (BMW and FAK) agreed on 59.0% (124/210) of the 210 posts (weighted *F_1_*-score=0.605; κ=0.344; Table 3). On the 200-post consensus-labeled subset, expert-to-consensus weighted *F*_1_-score values were 0.761 and 0.828, with κ values of 0.605 and 0.724. These comparisons describe different aspects of the annotation process and are not a single performance ceiling (Figure 2). In the fixed 150-post test set, the experts disagreed on 63 posts (42.0%). Gemini-3-Flash made 52 errors relative to the consensus reference, and 29 of those errors (55.8%) occurred in expert-disagreement cases.

**Table 3.** Agreement between human experts and the adjudicated reference labels^a^.

Comparison	Precision (weighted)	Recall (weighted)	*F*_1_-score (weighted)	Agreement (%)	Cohen κ
Expert 1 versus expert 2 (preadjudication)	0.649	0.591	0.605	59.05	0.344
Expert 1 versus expert 2 (consensus-labeled subset)	0.672	0.620	0.633	62.00	0.389
Expert 1 versus adjudicated consensus	0.764	0.765	0.761	76.50	0.605
Expert 2 versus adjudicated consensus	0.841	0.825	0.828	82.50	0.724

a The preadjudication comparison uses all 210 annotated posts. The consensus-labeled-subset and expert-to-consensus comparisons use the 200 posts with a majority consensus. Metrics are presented as contextual references and should not be interpreted as a single performance ceiling.

**Figure 2.**
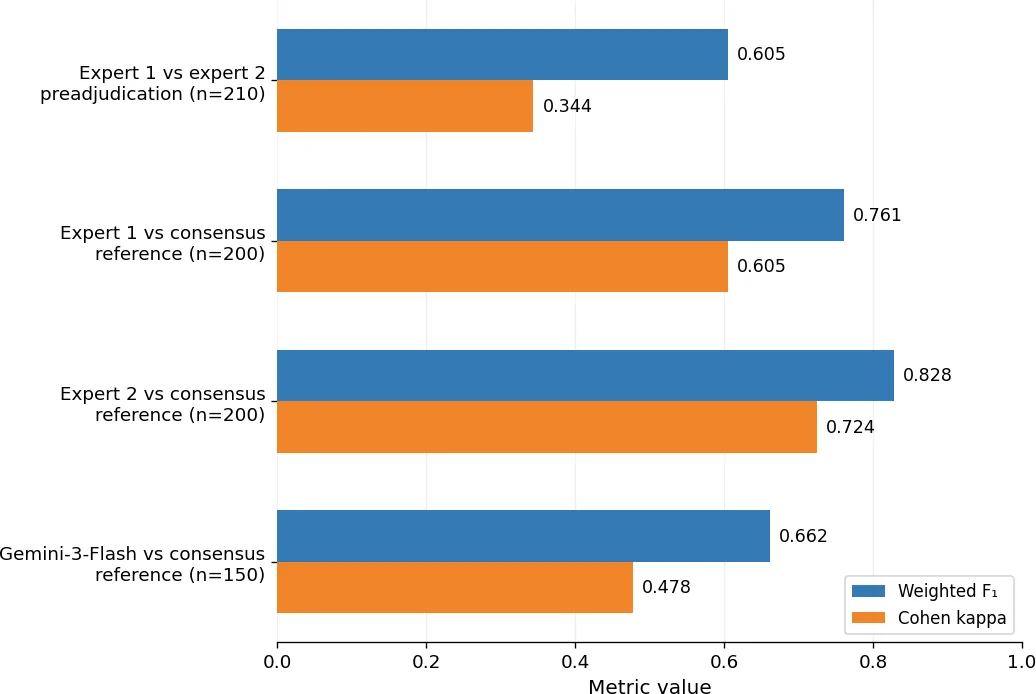
Weighted *F*_1_-score and Cohen κ for expert-expert, expert-consensus, and model-consensus comparisons. The expert-expert preadjudication comparison uses all 210 annotated posts; expert-consensus comparisons use the 200 consensus-labeled posts; and the Gemini-3-Flash comparison uses the fixed 150-post test set. The metrics contextualize agreement under different references and sample sizes and are not a direct inferential ranking.

Paraphrased examples from the annotated test set illustrate these boundaries. In one case, the writer reported substantial weight gain by pregnancy, noted that the clinician had not expressed concern, and asked whether the experience was typical; the primary experts differed between neutral and negative. In the second case, the writer celebrated a reassuring glucose-screening result while expressing distress about weight gain and urgency to lose it postpartum; the experts differed between positive and negative. In the third case, the writer described prolonged nausea and no net weight gain by the late second trimester and asked whether maternal and infant health could be affected; the experts differed between neutral and negative. These cases show how the assigned label can depend on whether the annotator prioritizes explicit wording, implied distress, or the dominant tone of the full narrative.

### Benchmarking Model Performance

Comparative performance on the fixed held-out test set (n=150) is summarized in Table 4. The table reports the selected prompting configuration for each generative model and the aligned-input direct-argmax configuration for each encoder model. Gemini-3-Flash had the numerically highest weighted *F*_1_-score point estimate (0.662; 95% CI 0.587‐0.729; κ=0.478), but CIs overlapped across the leading commercial, open-weight, and encoder models. Accordingly, these results do not establish statistically significant superiority of one model.

**Table 4.** Fixed held-out benchmark performance across model groups (n=150)^a^

Model family	Model	Configuration	Weighted precision (95% CI)	Weighted recall (95% CI)	Weighted *F*_1_-score (95% CI)	Cohen κ (95% CI)
Commercial LLM^b^	Gemini-3-Flash	0-shot	0.727 (0.664‐0.797)	0.653 (0.580‐0.727)	0.662 (0.587‐0.729)	0.478 (0.371‐0.580)
Commercial LLM	o3-Mini	5-shot	0.701 (0.629‐0.772)	0.620 (0.540‐0.700)	0.625 (0.545‐0.700)	0.435 (0.324‐0.547)
Commercial LLM	GPT-5-Mini^c^	5-shot	0.698 (0.632‐0.769)	0.613 (0.533‐0.687)	0.613 (0.530‐0.690)	0.430 (0.323‐0.537)
Encoder-only	BERTweet	Direct argmax	0.628 (0.547‐0.709)	0.640 (0.560‐0.707)	0.623 (0.539‐0.697)	0.369 (0.236‐0.484)
Encoder-only	RoBERTa	Direct argmax	0.632 (0.543‐0.721)	0.640 (0.560‐0.713)	0.605 (0.515‐0.692)	0.339 (0.211‐0.457)
Open-weight LLM	GPT-OSS	0-shot	0.666 (0.591‐0.750)	0.640 (0.560‐0.713)	0.645 (0.568‐0.721)	0.436 (0.316‐0.552)
Open-weight LLM	OpenChat	0-shot	0.632 (0.548‐0.714)	0.640 (0.560‐0.707)	0.627 (0.541‐0.703)	0.407 (0.289‐0.515)
Open-weight LLM	Mixtral	0-shot	0.634 (0.558‐0.714)	0.613 (0.540‐0.687)	0.620 (0.546‐0.694)	0.380 (0.264‐0.492)
Open-weight LLM	Gemma3	1-shot	0.654 (0.570‐0.739)	0.607 (0.527‐0.680)	0.611 (0.530‐0.687)	0.376 (0.257‐0.487)
Open-weight LLM	LLaMA3.1	0-shot	0.595 (0.502‐0.687)	0.620 (0.533‐0.700)	0.593 (0.498‐0.677)	0.322 (0.191‐0.437)

a Weighted precision, recall, *F*_1_-score, and Cohen κ are reported with 95% bootstrap CIs based on 1000 resamples. Generative-model configurations were selected on the validation set. RoBERTa and BERTweet use minimally processed text and direct argmax for the primary comparison.

b LLM: large language model.

c GPT: generative pretrained transformer.

Open-weight models produced point estimates in the same broad range as the commercially hosted models (Figure 3). GPT-OSS achieved a weighted *F*_1_-score=0.645 (κ=0.436), while OpenChat and Mixtral 8 × 7B achieved *F_1_*=0.627 and 0.620. Given the overlapping intervals, these differences are descriptive rather than a definitive ranking.

**Figure 3.**
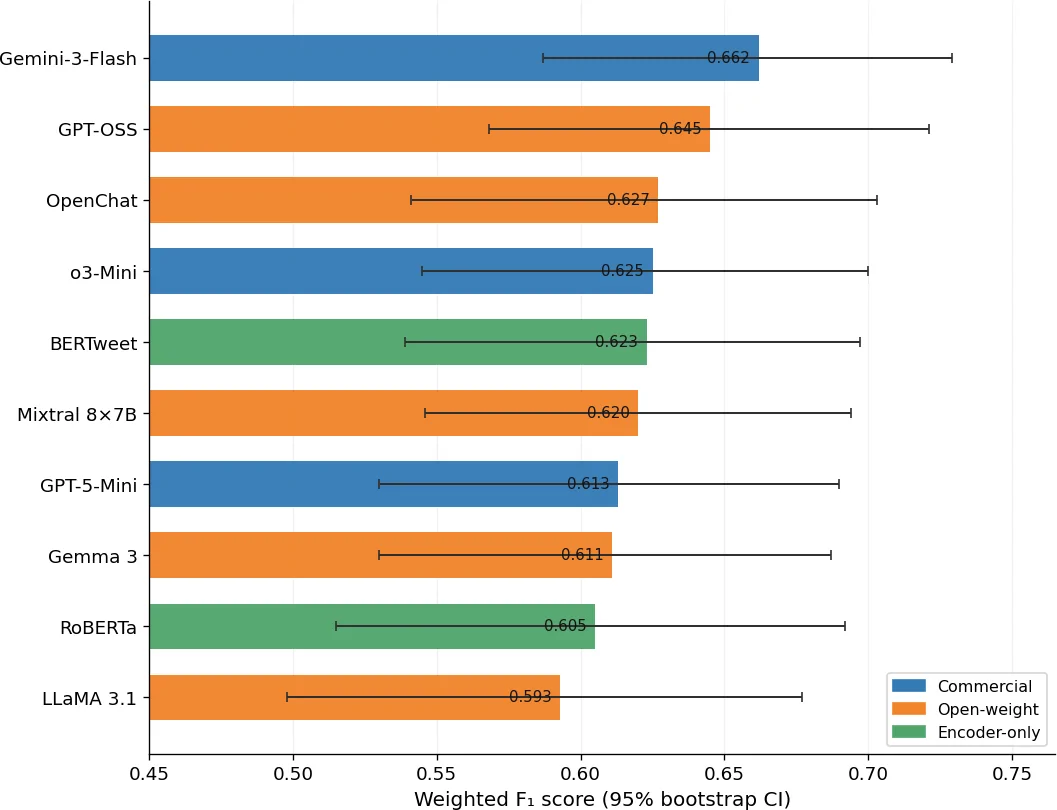
Weighted *F*_1_-score point estimates and 95% bootstrap CIs for the fixed held-out test set (n=150). Generative-model configurations were selected on the validation set. Encoder-only models use minimally processed text and direct argmax. Model groups are shown for descriptive comparison; the intervals overlap substantially. BERT: Bidirectional Encoder Representations from Transformers; GPT: generative pretrained transformer.

With aligned inputs and direct argmax, BERTweet achieved weighted *F*_1_-score=0.623 (κ=0.369), and RoBERTa achieved *F*_1_-score=0.605 (κ=0.339). Validation-calibrated results are reported in Supplementary Table S3 (Multimedia Appendix 1). In the separate task-adaptation analysis, fine-tuned RoBERTa achieved mean weighted *F*_1_-score=0.708 (SD 0.087; mean κ 0.534, SD 0.141), while fine-tuned BERTweet achieved mean *F*_1_-score=0.616 (SD 0.079; mean κ 0.385, SD 0.114; Supplementary Table S2 in Multimedia Appendix 1). These cross-validated estimates use all 200 consensus-labeled posts and are not directly ranked against the fixed held-out results.

### Error Analysis and Prompt Sensitivity

The Gemini-3-Flash confusion matrix showed that errors were concentrated at the neutral-negative boundary (Figure 4). Matter-of-fact descriptions of weight change were sometimes difficult to distinguish from narratives containing implicit distress, illustrating the limitations of a coarse 3-class polarity scheme for mixed-affect health text.

Prompt sensitivity was evaluated on the 40-post validation set using Gemini-3-Flash and GPT-OSS as representative commercially hosted and open-weight models (Table 5). Additional examples did not produce consistent improvement.

**Figure 4.**
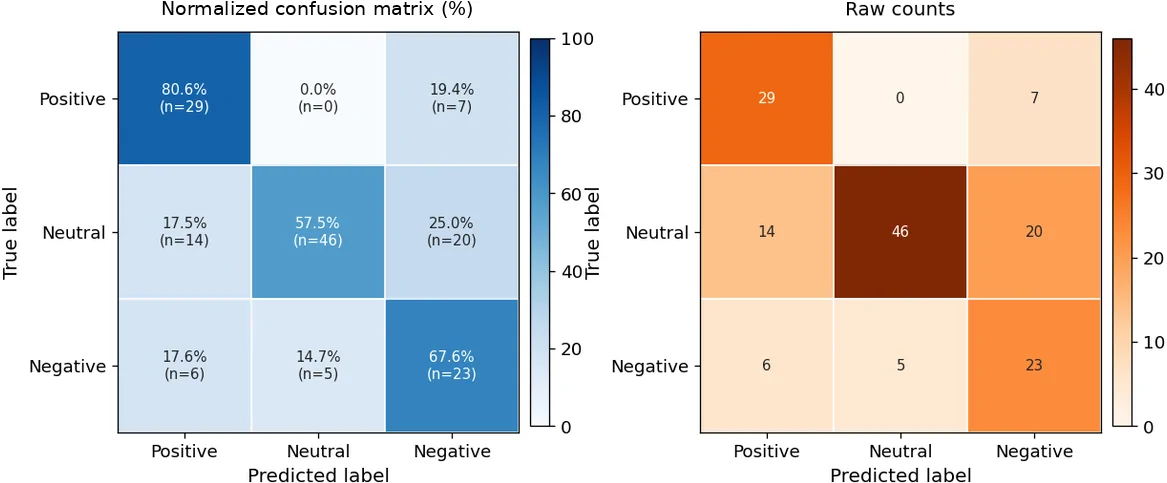
Confusion matrix for Gemini-3-Flash on the benchmark test set. Misclassifications are concentrated at the neutral-negative boundary, reflecting the challenge of distinguishing informational content from negatively valenced narratives.

**Table 5.** Weighted *F*_1_-score on the 40-post validation set under 0-shot, 1-shot, 3-shot, and 5-shot prompting for 2 representative instruction-following models.

Prompt strategy	Gemini-3-Flash	GPT-OSS^a^
0-shot	0.666	0.637
1-shot	0.646	0.582
3-shot	0.664	0.632
5-shot	0.608	0.632

a GPT: generative pretrained transformer.

For Gemini-3-Flash, 0-shot and 3-shot prompting produced similar weighted *F*_1_-score values (0.666 and 0.664), whereas the 5-shot configuration was lower (0.608). GPT-OSS also showed no consistent advantage from additional examples. These results indicate sensitivity to model and exemplar selection, but the small validation set does not support a firm causal explanation for the observed differences.

### Annotation Extension

Of the 150 posts sampled for the annotation extension, 106 met the study relevance criteria and received consensus sentiment labels. The 2 primary experts (BMW and FAK) agreed on 68/106 posts (64.2%; κ=0.367), with the remaining 38 disagreements reviewed by the third expert (RR).

On this extension, Gemini-3-Flash achieved weighted *F*_1_-score=0.633 (95% CI 0.522‐0.740; κ=0.374, 95% CI 0.233‐0.518), while GPT-OSS achieved weighted *F*_1_-score=0.680 (95% CI 0.580‐0.770; κ=0.428, 95% CI 0.289‐0.559). The CIs overlapped substantially. Model errors again concentrated in cases of primary-expert disagreement: 19/34 Gemini-3-Flash errors (55.9%) and 18/31 GPT-OSS errors (58.1%) occurred among posts on which the 2 primary experts initially disagreed.

In the exploratory negative-versus-nonnegative prioritization analysis, both models identified 56 of 58 expert-negative posts (96.6% sensitivity). Gemini-3-Flash flagged 85/106 posts (80.2%) for review, and GPT-OSS flagged 82/106 (77.4%), indicating high capture of expert-negative posts but only a modest reduction in review volume.

### Large-Scale Application to the Unlabeled Corpus

Gemini-3-Flash generated predictions for 400 unlabeled posts, and GPT-OSS generated predictions for 15,019 comments. These outputs were used to describe model-inferred patterns and are not expert-validated estimates of sentiment prevalence.

Model-inferred distributions are shown in Table 6 and Figure 5. Gemini-3-Flash classified 77.5% (310/400) of posts as negative, whereas GPT-OSS classified 40.3% (6048/15,019) of comments as positive. This apparent post-comment contrast should be interpreted as hypothesis-generating only and not as evidence of a true shift in sentiment between initiating posts and peer comments. The comparison simultaneously varies model and discourse type. Different models were used for posts and comments, comments were not expertly annotated, and intermodel agreement was low on the 1500-comment sample.

**Figure 5.**
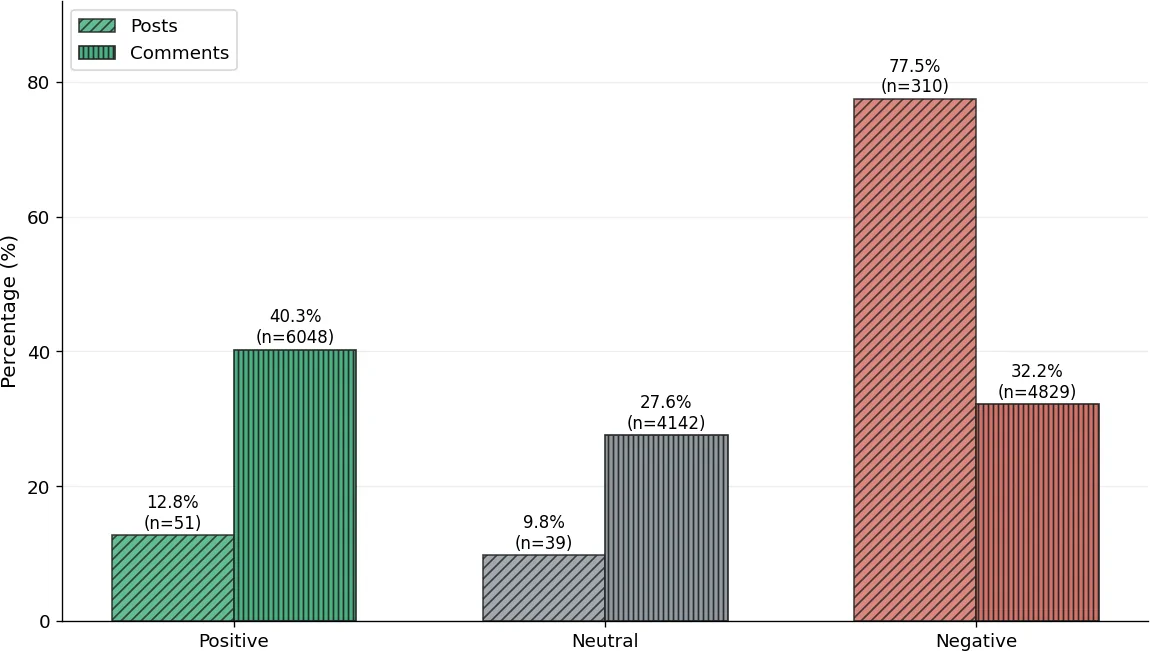
Model-inferred sentiment distributions for unlabeled posts and comments. Posts were classified with Gemini-3-Flash and comments with GPT-OSS. The contrast is exploratory because comments lacked expert labels, and the comparison combines model and discourse-type effects.

**Table 6.** Model-inferred sentiment distributions for 400 unlabeled posts and 15,019 comments^a^.

Sentiment	Posts, n (%)	Comments, n (%)	Difference (posts − comments, %)
Positive	51 (12.75)	6048 (40.27)	−27.52
Neutral	39 (9.75)	4142 (27.58)	−17.83
Negative	310 (77.50)	4829 (32.15)	+45.35
Total	400 (100.00)	15,019 (100.00)	0.00

a Posts were classified with Gemini-3-Flash and comments with GPT-OSS. Because model and discourse type differ simultaneously, the percentage differences are descriptive and cannot be attributed solely to discourse type.

To assess whether the observed distributions were consistent across models, Gemini-3-Flash and GPT-OSS were also compared on the same unlabeled texts. Across the 400 posts with valid paired predictions, the models agreed on 92.3% (369/400; κ=0.797). Agreement fell sharply to 46.3% (695/1500) for comments (κ=0.102; Figure 6).

These findings indicate that the predominantly negative post-level distribution was relatively consistent across the 2 models, whereas comment-level classifications were substantially more model-dependent. However, because neither unlabeled set had expert reference labels, these agreement estimates measure model concordance rather than classification accuracy or sentiment prevalence.

To examine a substantive downstream application, we compared valence-neutral pregnancy-weight discussion contexts among the 369 posts on which Gemini-3-Flash and GPT-OSS agreed. Explicit weight-gain or increase discussion occurred in 111/298 (37.2%) posts classified as negative compared with 7/71 (9.9%) posts classified as nonnegative (odds ratio =5.43, 95% CI 2.40‐12.26; FDR-adjusted q<0.001; Supplementary Table S6 in Multimedia Appendix 1). Other predefined discussion contexts did not remain significant after multiple-comparison correction. This exploratory result illustrates how sentiment classifications can be used to prioritize substantive contexts for qualitative follow-up rather than serving as stand-alone estimates of emotional state or population prevalence.

**Figure 6.**
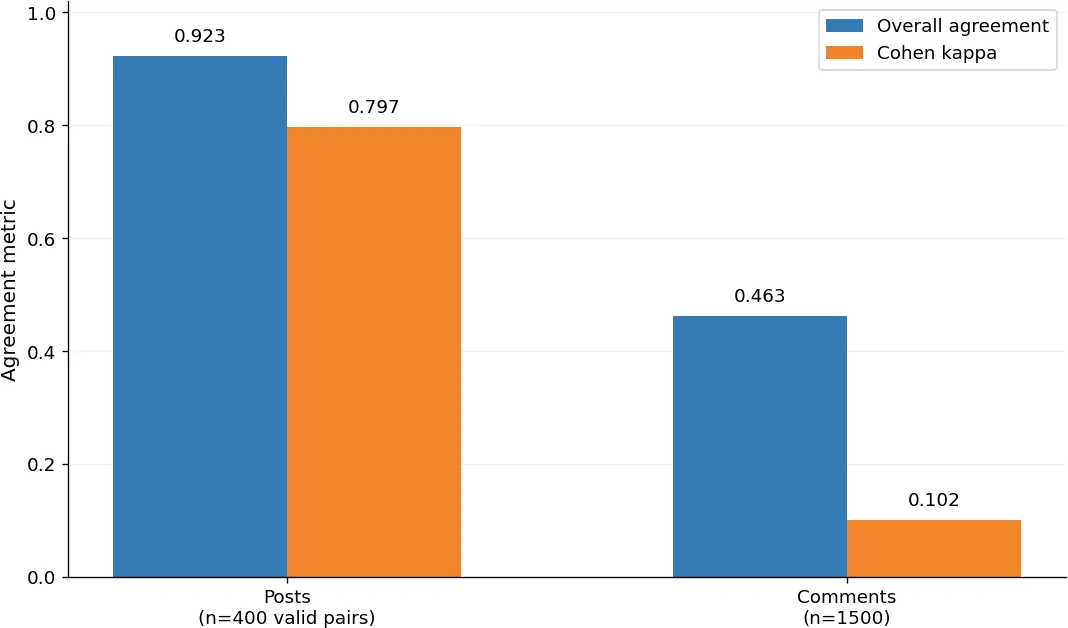
Intermodel agreement between Gemini-3-Flash and GPT-OSS during corpus-scale inference. Agreement is shown for 400 posts with valid paired predictions and a 1500-comment sample. High post-level agreement indicates consistency, not validated accuracy. Low comment-level agreement shows that model concordance on posts did not extend to comments.

## Discussion

### Overview

Online discussions of pregnancy-related weight provide a potentially valuable window into concerns that may not be fully expressed during clinical encounters, including uncertainty about normal weight gain, body-image distress, stigma, and perceived loss of control. However, using automated sentiment analysis to study these conversations requires confidence that models can interpret emotionally complex health narratives in ways that are sufficiently aligned with human judgment. In this study, no single model or model group consistently outperformed the others. Instead, performance was shaped by the availability of labeled data, the adaptation strategy, the ambiguity of the underlying text, and the type of discourse to which models were applied.

### Principal Findings

The principal contribution of this study is not a ranking of sentiment models or an estimate of sentiment prevalence, but an empirical definition of boundary conditions for broad sentiment classification in naturalistic maternal-health text. Across the analyses, broad sentiment was useful for corpus organization, review prioritization, and hypothesis generation, but insufficient without additional validation for inferring individual emotional state or population-level sentiment. The study therefore offers a framework for interpreting when sentiment analysis is informative in sensitive patient-generated health discourse and when richer, context-aware representations are needed.

Commercial, open-weight, and encoder models produced broadly overlapping CIs in the fixed held-out comparison. Gemini-3-Flash and GPT-OSS yielded weighted *F*_1_-score point estimates of 0.662 and 0.645, respectively; however, the results did not establish the superiority of any single model or model group. The separate cross-validation sensitivity analysis showed that supervised task adaptation materially improved RoBERTa (mean *F*_1_-score 0.708, SD 0.087). This finding indicates that relative model performance depends not only on architecture, but also on the availability of labeled data and the adaptation regime used. For maternal health researchers, this distinction is important because the choice of modeling approach should be informed by the intended application. The strong performance of task-adapted RoBERTa suggests that supervised adaptation may be advantageous when domain-specific labeled data are available. Conversely, the competitive performance of instruction-following LLMs without task-specific training suggests that they may be useful for exploratory analyses when rapid or resource-efficient deployment is needed.

The separate annotation extension provided a secondary check of the 2 models carried forward to post-level corpus inference. Their performance remained within the broad range observed in the primary benchmark, with overlapping uncertainty intervals, reinforcing the conclusion that small differences in point estimates should not be interpreted as evidence of clear model superiority.

The corpus-scale inference analysis further showed that model behavior varied by discourse type. Posts were more often classified as negative, whereas comments were more often classified as positive. At the post-level, Gemini-3-Flash and GPT-OSS demonstrated high concordance, suggesting that the broad distribution was relatively consistent across the 2 models. Conversely, agreement fell sharply when the models were applied to comments. The comment-level pattern therefore remains exploratory because comments lacked expert labels, were not used during prompt development or model validation, and were classified under a mixed model design.

The public-health significance of these findings lies not in identifying a single best-performing model, but in clarifying the conditions that should guide the responsible evaluation and deployment of automated sentiment classification. At scale, automated sentiment classification may eventually support the efficient analysis of large volumes of online maternal health discourse that would be difficult to review manually. At the performance levels observed here, however, these systems are currently better positioned to support human review and hypothesis generation than to independently classify sensitive health discourse for consequential applications such as surveillance or population comparison.

The RoBERTa adaptation findings also suggest that investment in domain-relevant labeled datasets may be particularly valuable. Fine-tuning materially improved RoBERTa within the separate cross-validation analysis, demonstrating that a comparatively compact encoder model can benefit substantially from examples drawn directly from the target discourse. Although the cross-validation estimates should not be directly compared with the fixed held-out benchmark because the evaluation designs differed, the findings nevertheless suggest that the availability of high-quality labeled data may be as important as the initial choice of model architecture.

These findings highlight 2 complementary deployment strategies. Instruction-following LLMs may offer a useful starting point when labeled data are limited and rapid exploratory classification is the primary goal. In contrast, when sustained application, local deployment, reproducibility, or greater control over the analytical pipeline is desired, developing an expert-labeled reference set and evaluating supervised task adaptation may be a better investment. Carefully developed reference sets may allow smaller or locally deployable systems to achieve competitive performance while providing additional advantages for privacy, reproducibility, cost, and institutional control over sensitive health data.

However, the ability to scale inference does not eliminate the need for context-specific validation. The findings from this study suggest that model performance is inherently dependent on the characteristics of the data used for development and evaluation, emphasizing that automated sentiment classification should be validated within the population, discourse type, and public-health context in which it will ultimately be applied.

### Human Disagreement as Evaluation Context

Model performance must be interpreted in the context of human disagreement. Our use of majority consensus is therefore best understood as an operational reference for conventional accuracy-based evaluation rather than evidence that each item has one objectively correct sentiment label. Preadjudication expert agreement (κ=0.344) and expert-to-consensus agreement quantify different aspects of the annotation process and should not be collapsed into a single numerical ceiling.

Disagreement should not be dismissed as annotation noise, but neither does it imply that all interpretations are equally valid. Instead, it may indicate that the 3-class task compresses clinically distinct states into a single dominant-polarity label. The dataset-derived examples above show that a numerical weight report may also function as reassurance-seeking, positive pregnancy news may coexist with weight-related distress, and a clinically framed account of inadequate gain may imply fear without using explicit emotion words. The finding that 55.8% (29/52) of Gemini-3-Flash errors occurred in expert-disagreement cases suggests that a substantial share of errors arose near these interpretive boundaries. It does not establish a human performance ceiling and reinforces the need to report consensus-based performance alongside annotator disagreement. The same pattern was observed in the annotation extension, where 55.9% (19/34) of Gemini-3-Flash errors and 58.1% (18/31) of GPT-OSS errors occurred in primary-expert disagreement cases.

The consistently modest agreement should not be attributed to a single source. It likely reflects interacting effects of the 3-class sentiment construct, the annotation protocol, and the complexity of patient-generated discourse. The task compresses heterogeneous experiences into one dominant-polarity label, while the available annotation protocol did not include a separately validated emotion codebook or formal calibration phase. Social media platforms such as Reddit can also provide spaces for open, patient-generated discussions in which factual clinical information may coexist with reassurance-seeking, body-image concerns, stigma, and fear. Model behavior and discourse type differences represent additional sources of uncertainty in automated classification. Because the current design cannot partition these contributions quantitatively, disagreement should not be interpreted as arising exclusively from either annotation quality or the Reddit data source.

For public health applications, this distinction is consequential. A post labeled negative may reflect transient frustration, clinically meaningful distress, body-image dissatisfaction, fear about pregnancy complications, or several of these experiences simultaneously. These experiences should not be treated as interchangeable, and their clinical or public-health significance cannot be recovered from a single polarity label. Accordingly, corpus-level sentiment distributions should not be interpreted as direct measures of patient distress or used to infer the prevalence of specific psychosocial concerns without additional validation. Instead, this methodology may be particularly valuable as a signal-detection tool. Sentiment patterns may help uncover patient experiences that are difficult to capture through traditional clinical data or encounters. These patterns can first identify areas of patient-generated discourse that warrant attention, after which qualitative examination of the underlying narratives can help determine what concerns or experiences those signals represent. These insights may reveal unmet informational needs or potential gaps in patient-provider communication and identify topics warranting greater attention in patient education or broader public health messaging. Future work may therefore benefit from moving beyond broad polarity toward multidimensional constructs such as intensity of distress, stigma, uncertainty, and perceived support. Future annotation studies should also retain richer annotator-level information and examine approaches that represent label distributions or multiple defensible interpretations rather than relying exclusively on a single aggregated consensus label.

Conversational dependence adds another challenge: a negatively valenced statement may express distress or support-seeking and subsequently elicit reassurance or normalization, so its interactional meaning may not be captured by isolated polarity. This is particularly important when research questions examine peer support or other interactional processes, for which classifying post replies independently may obscure the function they serve within the broader conversation. Future work should therefore evaluate conversation-aware annotation and modeling that preserves parent-reply relationships.

### Architecture, Adaptation, and Neutral-Negative Ambiguity

Error concentration at the neutral-negative boundary (Figure 4) suggests that “Neutral” often functions as a category for informational, mixed, or implicitly distressed narratives rather than as a simple sentiment midpoint. Models identified overt affect more consistently but struggled when negative valence was embedded within clinical descriptions or body-image concerns. This pattern highlights the limitations of 3-class polarity taxonomy for affectively complex health narratives.

The analyses do not support a claim that one architecture family is inherently superior. Off-the-shelf point estimates clustered within overlapping intervals, while supervised fine-tuning materially improved RoBERTa but not BERTweet to the same extent. The practical comparison is therefore between deployment regimes. Instruction-following LLMs can provide competitive zero- or few-shot performance without task-specific training and may be suitable for initial feasibility studies or rapidly changing research questions. Encoder models require labeled examples and additional training, but may perform strongly when labeled data and fine-tuning resources are available. For LLMs, prompt sensitivity also remained model dependent, with no uniform benefit from adding examples. Notably, few-shot prompting often degraded performance relative to zero-shot configurations, highlighting a lack of robustness in in-context learning for emotionally heterogeneous health text. For maternal health sentiment, zero-shot inference may offer a more stable and reproducible pipeline.

### Implications for Scalable Digital Health Research

Inter-model agreement differed sharply by discourse type (Figure 6). Gemini-3-Flash and GPT-OSS agreed on 92.3% (369/400) of the 400 valid post pairs (κ=0.797), but on only 46.3% (695/1500) of the 1500 comments (κ=0.102). Because neither unlabeled set had expert reference labels, these values measure consistency between models rather than the validity or accuracy of either model’s predictions.

This contrast likely reflects fundamental differences between initiating posts and peer responses. The benchmarked initiating posts and unlabeled posts consisted of comparatively longer, self-contained narratives describing pregnancy-related weight experiences. Comments, by contrast, were often shorter, conversational, and largely dependent on the content of the initiating post or preceding replies. Applying models validated on initiating posts to comments may therefore introduce errors arising from missing conversational context, discourse-type differences, or model-specific classification behavior.

From a public health perspective, initiating posts and peer responses should therefore be treated as distinct analytical targets rather than interchangeable units of social-media text. Individuals may begin discussions to express uncertainty, seek advice, describe pregnancy-related weight changes, or share feelings of distress or frustration, whereas responses may provide reassurance, normalize pregnancy experiences, share lived experiences, recount a different lived experience, challenge the premise of the post, or respond to an earlier comment rather than to the initiating narrative. If these communicative functions differ systematically, models validated only on initiating narratives may inadequately characterize the supportive interactions that make online communities valuable sources of maternal health information and peer support.

These findings have important implications for the responsible use of automated sentiment analysis in digital public health research. High agreement between Gemini-3-Flash and GPT-OSS on initiating posts suggests that open-weight alternatives may warrant consideration when deployment data closely resemble the text used for validation. However, concordance between models does not establish accuracy or interchangeability, and model selection should continue to consider uncertainty, reproducibility, computational requirements, and cost. Sentiment models may also support exploratory assessment of how patterns in online discourse change following new clinical guidance or public health messaging. At present, these classifications are best viewed as complementary to, rather than replacements for, expert interpretation.

Furthermore, the exploratory corpus analysis provides a complementary example. Negative model-consensus sentiment was disproportionately concentrated in posts explicitly discussing weight gain or increase. Although this finding does not establish clinical distress or population prevalence, it illustrates how sentiment classification can be combined with transparent content indicators to locate potentially meaningful patterns for subsequent qualitative or epidemiologic investigation.

The value of Reddit in this setting is not as a representative measure of sentiment, but as a source of unsolicited, naturalistic narratives about pregnancy-related weight, body image, stigma, uncertainty, counseling, and peer support outside structured clinical or research encounters. These data can complement surveys, interviews, and clinical records by surfacing language and concerns that inform qualitative coding, survey development, communication research, and epidemiologic hypotheses. At the same time, context-rich and mixed-affect narratives can make a single polarity label a lossy representation. Modest sentiment agreement therefore does not imply that the narratives lack research value; rather, it indicates that richer, context-aware constructs may better capture that value.

Taken together, these findings support a staged approach to automated analysis of sensitive patient-generated health discourse. Researchers should determine whether broad sentiment is an adequate construct for the intended question, establish an expert-labeled reference set that preserves ambiguity, compare prompted and supervised approaches under clearly separated evaluation designs, and validate the selected model on each discourse type to which it will be applied. Corpus-scale outputs should then be used to guide human interpretation and hypothesis development. Under this framework, automated sentiment analysis has the potential to make large and otherwise difficult-to-review collections of patient-generated text more accessible for rigorous, context-sensitive investigation.

### Strengths and Limitations

Several limitations bound the generalizability of the findings. The corpus was limited to English-language Reddit content and may not represent pregnancy-related experiences on other platforms, in other languages, or among populations that do not use social media for pregnancy-related discourse. The corpus should not be interpreted as representative of pregnant individuals generally. Reddit search may introduce undocumented ranking, indexing, query-limit, rate-limit, and historical-coverage biases, and user geography and other relevant demographic characteristics were unavailable. Complete post-ID recovery through Arctic Shift supports cross-source reproducibility of the observed retrieval but does not establish that either source represents a complete historical census of relevant Reddit posts. The primary consensus benchmark remained modest (n=200) and therefore limited statistical precision for resolving small differences among models. The additional 106-post expert-annotated extension increased labeled coverage and provided a separate secondary evaluation of the 2 corpus-scale post classifiers but does not replace the need for larger and more diverse reference sets. The 3-class framework also compresses clinically and socially distinct experiences into broad polarity labels. A negative label may reflect transient frustration, body-image dissatisfaction, fear of complications, shame, stigma, guilt, ambivalence, or perceived loss of control, which differ in clinical and public-health meaning. Comment-level findings were particularly limited because comments lacked expert labels and the comparison simultaneously varied model and discourse type. These limitations also prevent quantitative decomposition of the sources of disagreement, which likely include the sentiment construct, annotation protocol, mixed-affect narrative content, discourse characteristics, and model behavior. Future studies could separate these components by comparing calibrated annotation rubrics, single- versus multilabel or dimensional emotion representations, and isolated versus conversation-aware text.

The study nevertheless has several strengths. It uses expert adjudication while preserving and reporting disagreement rather than treating consensus labels as unquestioned ground truth. It also separates off-the-shelf held-out benchmarking from supervised task adaptation, reducing the risk of conflating model architecture with adaptation regime. The inclusion of commercially hosted, locally deployable, and encoder-only systems reflects practical options available to public-health and health-informatics researchers operating under differing data, privacy, cost, and computational constraints. Finally, the post-comment analysis directly examines the limitations of transferring inference across discourse types. These findings provide a transparent formative assessment of where broad sentiment classification can support scalable analysis of patient-generated health text, where its interpretation becomes unstable, and where richer annotation or conversational context is required.

### Conclusion

This formative benchmark demonstrates that sentiment classification performance in pregnancy-related weight discourse depends on human interpretive ambiguity, access to labeled data, the adaptation regime, and the discourse type under analysis. The fixed held-out comparison did not establish a uniformly superior model or model group. In a separate cross-validated analysis, task-specific fine-tuning materially improved RoBERTa performance, while instruction-following LLMs provided competitive performance without task-specific training.

The findings underscore that model performance should not be interpreted independently of the reference-label process or deployment context. Human disagreement and metric uncertainty should be reported alongside model performance metrics, off-the-shelf and task-adapted evaluations should remain analytically distinct, and models should be validated before deployment across new discourse types. High intermodel agreement on unlabeled posts indicated consistency but not validated accuracy, and this concordance did not extend to comments. The annotation extension similarly showed that model errors remained concentrated in cases of expert disagreement and that high-sensitivity prioritization of expert-negative posts was possible, although the resulting reduction in human-review volume was modest.

Taken together, this study defines practical boundary conditions for sentiment analysis of naturalistic maternal-health discourse. Reddit offers valuable patient-generated narratives that may be difficult to capture in structured clinical data, but a single polarity label may not adequately summarize every narrative or conversational context. When used as a signal-detection tool instead of a stand-alone measure of patient experience, sentiment classification may help surface patterns that warrant closer qualitative investigation and, in turn, inform patient-provider communication, patient education, and broader public health messaging. Broad sentiment classification is therefore best used for corpus organization, expert-review prioritization, and hypothesis generation rather than as a stand-alone measure of emotional state or population sentiment. Future work should emphasize multidimensional, annotator-aware, and conversation-aware representations.

## Supplementary material

10.2196/92821 Multimedia Appendix 1 Supplementary textbox containing expanded Reddit search terms and 6 supplementary tables covering per-class model performance, fine-tuning and decision-rule sensitivity analyses, model identifiers and configurations, annotation-extension evaluation, and exploratory discussion-context results.

## Abbreviations

BERTweet: Bidirectional Encoder Representations from Transformers for Tweets
GPT: generative pretrained transformer
GWG: gestational weight gain
IOM: Institute of Medicine
LBW: low birth weight
LLM: large language model
NHSR: Not Human Subjects Research
NLP: natural language processing
PRAW: Python Reddit API Wrapper
RoBERTa: Robustly Optimized Bidirectional Encoder Representations from Transformers Approach
UTC: Coordinated Universal Time
UTHSC: University of Tennessee Health Science Center
