# Supplementary material for: Benchmarking Large Language Models for Sentiment Analysis in Pregnancy-Related Weight Discussions on Reddit: Human Performance-Aligned Comparative Evaluation Study
Source: JMIR Form Res. 2026 Nov 13;10:e92821. doi: 10.2196/92821 (PMC13766661; doi:10.2196/92821)
Supplement: Supplementary file 1 [file formative-v10-e92821-s001.docx]

**MULTIMEDIA APPENDIX 1**

**Supplementary Textbox S1.** Full list of expanded keywords generated via LLM (GPT-4.5).

pregnancy weight, weight gain pregnancy, extra weight when pregnant, gaining weight while pregnant, pregnant weight gain, gaining baby weight, extra pounds during pregnancy, pregnancy pounds, feeling fat while pregnant, feeling overweight when pregnant, pregnancy weight issues, weight struggles during pregnancy, struggling with pregnancy weight, pregnancy weight problems, overweight while pregnant, pregnant and overweight, pregnancy body changes, body changes during pregnancy, pregnancy body, pregnancy size, body image during pregnancy, pregnancy body image, pregnancy self-esteem, feeling self-conscious during pregnancy, pregnancy weight story, pregnancy weight journey, real pregnancy weight, honest pregnancy weight, pregnancy weight experiences, pregnancy weight diary, pregnancy weight frustrations, pregnancy weight complaints, weight worries during pregnancy, pregnancy weight concerns, am I gaining too much weight during pregnancy, too many pounds when pregnant, pregnancy weight gain tips, pregnancy diet tips, diet for pregnant women, healthy eating during pregnancy, eating habits during pregnancy, pregnancy food cravings, cravings during pregnancy, eating too much while pregnant, overeating during pregnancy, calorie intake when pregnant, bloating during pregnancy, pregnancy bloating, water retention during pregnancy, swollen during pregnancy, pregnancy body issues, body shaming during pregnancy, feeling judged about my pregnancy weight, shamed for pregnancy weight, pregnancy weight stigma, body confidence during pregnancy, pregnancy body confidence, feeling unattractive when pregnant, pregnancy weight insecurities, negative body image pregnancy, positive body image pregnancy, pregnancy weight support, help with pregnancy weight, losing weight while pregnant, managing weight during pregnancy, weight management during pregnancy, pregnancy fitness, exercise during pregnancy, staying fit while pregnant, pregnancy weight loss advice, postpartum weight, bouncing back after pregnancy, recovery weight after birth, pregnancy belly fat, stubborn pregnancy weight, excess weight during pregnancy, health concerns with pregnancy weight, pregnancy weight and health, pregnancy weight complications, trouble with pregnancy weight, difficult pregnancy weight, struggling with body image when pregnant, society and pregnancy weight, cultural views on pregnancy weight, opinions on pregnancy weight, talking about pregnancy weight, discussion on pregnancy weight, pregnancy weight discussion, pregnancy weight rant, venting about pregnancy weight, pregnant and feeling heavy, too many pounds in pregnancy, excess pounds during pregnancy, unhappy with my pregnancy weight, pregnancy weight pressure, social media and pregnancy weight, online discussion pregnancy weight, pregnancy weight advice, advice on gaining weight during pregnancy, pregnancy weight help, pregnancy weight check, scale shock during pregnancy, pregnancy and body changes, pregnancy expectations vs reality, my pregnancy journey, pregnancy transformation, pregnancy body transformation, changes in my body during pregnancy, weight gain experience pregnancy, pregnancy weight gain experience, pregnancy weight testimonial, pregnancy weight review, pregnancy and weight challenges, pregnancy struggles with weight, mom-to-be weight issues, expecting weight issues, weight gain and pregnancy, pregnancy weight reaction, difficulties with pregnancy weight, coming to terms with pregnancy weight, being pregnant and overweight, navigating pregnancy weight, pregnancy weight reality, challenge of pregnancy weight, balancing weight during pregnancy, getting used to pregnancy weight, managing extra weight in pregnancy, coping with pregnancy weight, accepting pregnancy weight, talk about being heavy while pregnant, working out during pregnancy, pregnancy exercise, pregnancy healthy weight, maintaining weight in pregnancy, self-care during pregnancy weight challenges, pregnancy eating challenges, pregnancy cravings and weight, relationship with food while pregnant, weight gain concerns in pregnancy, pregnancy weight and mental health, pregnancy mood and weight, emotional eating during pregnancy, pregnancy eating habits, weight talk in pregnancy

**Supplementary Table S1.** Per-class *F_1_*-scores for all evaluated models in the fixed held-out comparison (n = 150). Generative-model configurations reflect the selected zero- or few-shot setting. RoBERTa and BERTweet are reported using minimally processed text and direct argmax. (Table 4).

| **Model** | **Configuration** | ***F_1_* (Positive)** | ***F_1_* (Negative)** | ***F_1_* (Neutral)** | ***F_1_* (Weighted)** |
| --- | --- | --- | --- | --- | --- |
| Gemini-3-Flash | k0 | 0.682 | 0.548 | 0.702 | 0.662 |
| GPT-OSS | k0 | 0.642 | 0.533 | 0.694 | 0.645 |
| OpenChat | k0 | 0.674 | 0.364 | 0.717 | 0.627 |
| o3-Mini | k5 | 0.607 | 0.595 | 0.646 | 0.625 |
| Mixtral | k0 | 0.551 | 0.481 | 0.711 | 0.62 |
| GPT-5-Mini | k5 | 0.622 | 0.605 | 0.613 | 0.613 |
| Gemma3 | k1 | 0.517 | 0.538 | 0.685 | 0.611 |
| BERTweet | argmax, minimal | 0.588 | 0.407 | 0.730 | 0.623 |
| RoBERTa | argmax, minimal | 0.500 | 0.367 | 0.754 | 0.605 |
| llama3-1 | k0 | 0.379 | 0.441 | 0.754 | 0.593 |

**Supplementary Table S2.** Task-adaptation sensitivity analysis for RoBERTa and BERTweet using stratified 5-fold cross-validation across the 200 consensus-labeled posts. Each fold used 160 posts for training and 40 for evaluation. Values are mean (SD) across folds and are not directly comparable to the fixed held-out estimates in Table 4.

| **Model** | **Max length** | **Precision** | **Recall** | **Weighted *F_1_*** | **Accuracy** | **Cohen's κ** |
| --- | --- | --- | --- | --- | --- | --- |
| RoBERTa | 256 | 0.725 (0.095) | 0.710 (0.084) | 0.708 (0.087) | 0.710 (0.084) | 0.534 (0.141) |
| BERTweet | 128 | 0.615 (0.087) | 0.625 (0.071) | 0.616 (0.079) | 0.625 (0.071) | 0.385 (0.114) |

**Supplementary Table S3.** Encoder input and decision-rule sensitivity on the fixed held-out test set (n = 150). All rows use minimally processed text. Direct argmax is the primary comparison; validation-calibrated thresholds are shown as a sensitivity analysis.

| **Model** | **Decision rule** | **Precision** | **Recall** | **Weighted *F_1_*** | **Accuracy** | **Cohen's κ** |
| --- | --- | --- | --- | --- | --- | --- |
| RoBERTa | Direct argmax | 0.632 | 0.640 | 0.605 | 0.640 | 0.339 |
| RoBERTa | Validation calibrated | 0.621 | 0.620 | 0.576 | 0.620 | 0.284 |
| BERTweet | Direct argmax | 0.628 | 0.640 | 0.623 | 0.640 | 0.369 |
| BERTweet | Validation calibrated | 0.616 | 0.627 | 0.617 | 0.627 | 0.363 |

**Supplementary Table S4.** Model identifiers, access details, deployment settings, and evaluation configurations for the 10 models included in the benchmark. The benchmark configuration indicates the validation-selected prompt setting for generative models or the primary decision rule used for encoder-only classifiers.

| **Model group** | **Display name** | **Provider** | **API model string** | **Access route** | **Access date** | **Benchmark**  **configuration** |
| --- | --- | --- | --- | --- | --- | --- |
| Commercial LLM | GPT-5-Mini | OpenAI | "gpt-5-mini" | OpenAI v2.8.0; cloud | December 10, 2025 | 5-shot |
| Commercial LLM | o3-Mini | OpenAI | "o3-mini" | OpenAI v2.8.0; cloud | December 15, 2025 | 5-shot |
| Commercial LLM | Gemini-3-Flash | Google | "gemini-3-flash-preview" | Google-genai v1.56.0; cloud | December 17, 2025 | 0-shot |
| Open-weight LLM | LLaMA 3.1 (8B) | Meta | "llama3.1:8b" | Ollama v0.13.4; local | November 28, 2025 | 0-shot |
| Open-weight LLM | Gemma 3 (12B) | Google | "gemma3:12b" | Ollama v0.13.4; local | November 28, 2025 | 1-shot |
| Open-weight LLM | Mixtral (8x7B) | Mistral AI | "mixtral" | Ollama v0.13.4; local | November 28, 2025 | 0-shot |
| Open-weight LLM | OpenChat (7B) | OpenChat team | "openchat" | Ollama v0.13.4; local | November 28, 2025 | 0-shot |
| Open-weight LLM | GPT-OSS (20B) | OpenAI | "gpt-oss" | Ollama v0.13.4; local | December 08, 2025 | 0-shot |
| Encoder-only classifier | RoBERTa | CardiffNLP | cardiffnlp/twitter-roberta-base-sentiment-latest | Hugging Face Transformers v4.57.3 | July 08, 2026 | Primary: direct argmax. Sensitivity: calibrated thresholds and stratified 5-fold fine-tuning. |
| Encoder-only classifier | BERTweet | Finite-automata | finiteautomata/bertweet-base-sentiment-analysis | Hugging Face Transformers v4.57.3 | July 08, 2026 | Primary: direct argmax. Sensitivity: calibrated thresholds and stratified 5-fold fine-tuning. |

**Supplementary Table S5.** Annotation extension and secondary evaluation of Gemini-3-Flash and GPT-OSS. Bootstrap confidence intervals are based on 1,000 resamples.

| **Measure** | **Extension / Reference** | **Gemini-3-Flash** | **GPT-OSS** |
| --- | --- | --- | --- |
| Candidate extension posts | 150 | - | - |
| Retained after expert relevance screening | 106 | - | - |
| Primary-expert exact agreement | 68/106 (64.2%) | - | - |
| Primary-expert Cohen’s κ | 0.367 | - | - |
| Final consensus distribution | 58 Negative; 40 Neutral; 8 Positive | - | - |
| Weighted precision (95% CI) | - | 0.748 (0.661-0.820) | 0.761 (0.693-0.828) |
| Weighted recall (95% CI) | - | 0.679 (0.585-0.764) | 0.708 (0.623-0.792) |
| Weighted *F_1_* (95% CI) | - | 0.633 (0.522-0.740) | 0.680 (0.580-0.770) |
| Accuracy (95% CI) | - | 0.679 (0.585-0.764) | 0.708 (0.623-0.792) |
| Cohen’s κ (95% CI) | - | 0.374 (0.233-0.518) | 0.428 (0.289-0.559) |
| Expert-Negative sensitivity | - | 56/58 (96.6%) | 56/58 (96.6%) |
| Specificity | - | 39.6% | 45.8% |
| Positive predictive value | - | 65.9% | 68.3% |
| Negative predictive value | - | 90.5% | 91.7% |
| Binary F1 | - | 0.783 | 0.800 |
| Posts flagged for review | - | 85/106 (80.2%) | 82/106 (77.4%) |
| Errors in primary-expert disagreement cases | - | 19/34 (55.9%) | 18/31 (58.1%) |

**Supplementary Table S6.** Exploratory valence-neutral discussion-context comparison among the 369 posts with concordant Gemini-3-Flash and GPT-OSS predictions. Negative versus non-Negative comparisons use Fisher exact tests with Benjamini-Hochberg false-discovery-rate correction.

| **Discussion context** | **Negative n/N (%)** | **Non-Negative**  **n/N (%)** | **Difference (pp)** | **OR (95% CI)** | **FDR q** |
| --- | --- | --- | --- | --- | --- |
| Weight gain or increase | 111/298 (37.2%) | 7/71 (9.9%) | 27.4 | 5.43 (2.40-12.26) | <0.001 |
| Body size, appearance, or clothing | 111/298 (37.2%) | 18/71 (25.4%) | 11.9 | 1.75 (0.97-3.13) | 0.358 |
| Provider or weight monitoring | 52/298 (17.4%) | 7/71 (9.9%) | 7.6 | 1.93 (0.84-4.46) | 0.497 |
| Weight loss or low gain | 59/298 (19.8%) | 17/71 (23.9%) | -4.1 | 0.78 (0.42-1.45) | 0.912 |
| Fetal growth or measurement | 19/298 (6.4%) | 6/71 (8.5%) | -2.1 | 0.74 (0.28-1.92) | 0.912 |
| Peer information seeking | 82/298 (27.5%) | 17/71 (23.9%) | 3.6 | 1.21 (0.66-2.20) | 0.912 |
| Nutrition, eating, or cravings | 97/298 (32.6%) | 25/71 (35.2%) | -2.7 | 0.89 (0.52-1.53) | 0.912 |
| Postpartum weight or body change | 53/298 (17.8%) | 11/71 (15.5%) | 2.3 | 1.18 (0.58-2.40) | 0.912 |
| Symptoms affecting intake or weight | 56/298 (18.8%) | 12/71 (16.9%) | 1.9 | 1.14 (0.57-2.26) | 0.961 |
| Exercise or physical activity | 83/298 (27.9%) | 19/71 (26.8%) | 1.1 | 1.06 (0.59-1.89) | 1.000 |
